# Heat-Related Emergency Department Visits — United States, May–September 2023

**DOI:** 10.15585/mmwr.mm7315a1

**Published:** 2024-04-18

**Authors:** Ambarish Vaidyanathan, Abigail Gates, Claudia Brown, Emily Prezzato, Aaron Bernstein

**Affiliations:** ^1^Division of Environmental Health Science and Practice, National Center for Environmental Health, CDC; ^2^Detect and Monitor Division, Office of Public Health Data, Surveillance, and Technology, CDC; ^3^National Center for Environmental Health, Agency for Toxic Substances and Disease Registry, CDC.

**Summary**

**What is already known about this topic?**

Unprecedented heat waves can affect all persons, but some are more sensitive to the effects of heat, including children and adults with underlying health conditions, pregnant women, and outdoor workers.

**What is added by this report?**

During the 2023 warm-season months (May–September), rates of emergency department visits for heat-related illness substantially increased across several U.S. regions compared with previous years, especially among males and adults aged 18–64 years.

**What are the implications for public health practice?**

Heat-related illness will continue to be a significant public health concern as climate change results in longer, hotter, and more frequent episodes of extreme heat. By monitoring heat-related health impacts, public health agencies can detect trends in health care utilization rates, identify subpopulations at increased risk, and guide public health actions tailored to specific heat exposure levels.

## Abstract

Unprecedented heat waves can affect all persons, but some are more sensitive to the effects of heat, including children and adults with underlying health conditions, pregnant women, and outdoor workers. Many regions of the United States experienced record-breaking high temperatures in 2023, with populations exposed to extremely high temperatures for prolonged periods. CDC examined emergency department (ED) visits associated with heat-related illness (HRI) from the National Syndromic Surveillance Program and compared daily HRI ED visit rates during the warm-season months (May–September) of 2023 with those during 2018–2022. In the 2023 warm-season months, daily HRI ED visit rates peaked in several regions and remained elevated for a prolonged duration. More males than females sought care in EDs for HRI, especially males aged 18–64 years. CDC issued multiple public health alerts using the Epidemic Information Exchange system to bring attention to increases in ED utilization for HRI. Deaths and illnesses associated with heat exposure are a continuing public health concern as climate change results in longer, hotter, and more frequent episodes of extreme heat. Near real-time monitoring of weather conditions and adverse health outcomes can guide public health practitioners' timing of risk communication and implementation of prevention measures associated with extreme heat.

## Introduction

The warm-season months (May–September) of 2023 were the hottest ever recorded in the United States,* and adverse health impacts, including deaths and illnesses attributable to high ambient temperatures, received considerable attention.^†^ Hot weather conditions can affect all persons; however, for certain specific populations, exposure and health risks are compounded by adverse physiologic, behavioral, demographic, or socioeconomic factors that result in their being disproportionately affected by extreme heat (*1*). Populations at highest risk typically include older persons, children and adolescents, persons with preexisting health conditions, pregnant women, outdoor workers, persons with limited access to cooling resources, and persons living in low-income communities.^§^ Further, exceptionally hot conditions can increase the demand for medical services and strain health systems (e.g., a surge in persons seeking emergency department [ED] care) (*2*). Successful public health measures to reduce heat-related illness (HRI), including targeted communication and outreach campaigns for populations at risk, require coordination across various health care sectors and are often guided by near real-time assessments of heat exposure and its resulting adverse health impacts. To assess the health impact of exceedingly high temperatures observed during the warm-season months of 2023, CDC analyzed National Syndromic Surveillance Program (NSSP) data to compare daily HRI ED visit rates during May–September 2023 with those during May–September 2018–2022.

## Methods

### Data Sources

Data on HRI ED visits^¶^ occurring during January 2018–December 2023 were extracted from NSSP’s Electronic Surveillance System for the Early Notification of Community-Based Epidemics (ESSENCE).** The daily number of HRI and all-cause ED visits were tabulated for each of the 10 U.S. Department of Health and Human Services (HHS) regions.^††^ NSSP data were analyzed to compare the 2023 heat season with the 2018–2022 seasons. To account for temporal changes among facilities sharing data with NSSP, comparisons between 2023 and previous years were restricted to those EDs with consistent reporting during the study period.^§§^

### Descriptive and Statistical Analyses

After applying data quality filters to reduce artifactual changes in reporting patterns during 2018–2023, a maximum of 826 (range = 3–826; median = 36) ED facilities that participate in NSSP reported one or more visits associated with HRI. The HHS region–specific daily HRI ED visit rate (the number of ED visits for HRI per 100,000 all-cause ED visits) observed during the warm-season months of 2023 was compared with the 95th percentile value of the daily HRI ED visit rate distribution. The 95th percentile for each region was computed based on HRI ED data recorded for the 2018–2022 warm-season months. 

Differences in HRI ED visit rates were evaluated by age group (0–17, 18–25, 26–54, 55–64, 65–74, and ≥75 years), sex, HHS region, and occurrence of the HRI ED visits during the hotter warm-season months (i.e., July and August). Rate ratios (RRs) and associated 95% CIs were estimated using a multivariate Poisson regression model. The daily number of HRI visits was regressed against predictors such as age group, sex, HHS region, and an indicator to denote the occurrence of HRI ED visits during the hotter warm-season months of July and August. The model also included the logarithm of all-cause ED visits to account as an offset term. For each predictor, the category with the lowest warm-season HRI ED visit rate was identified as the referent population. Regressions were executed for 2023 and 2018–2022 with the same model specifications and parameters. Analysis and visualization were conducted using R software (version 4.1.2; R Foundation) and SAS software (version 9.4; SAS Institute). This activity was reviewed by CDC, deemed not research, and was conducted consistent with applicable federal law and CDC policy.^¶¶^

## Results

### Characteristics of HRI ED Visits

During January 1–December 31, 2023, a total of 119,605 HRI ED visits were recorded in the ESSENCE system***; 92% of these visits occurred during May–September. Across the study period, July and August accounted for a higher average HRI ED visit rate (303 per 100,000 ED visits) compared with other warm-season months (May, June, and September) (97) (Table 1). Further, the risk observed during July–August 2023 was more than three times that during May, June, and September (mean RR = 3.07), consistent with record-breaking temperatures observed across several HHS regions in 2023.^†††^ In comparison, the risk observed in July–August 2018–2022 was approximately twice as high as that of May, June, and September of the same period.

**TABLE 1:** Comparison* of mean rate and rate ratios for heat-related illness emergency department visits^†^ for warm-season months (May–September), by age group, sex, U.S. Department of Health and Human Services region,^§^ and peak heat season — United States, 2023 and 2018–2022

Characteristic	Year					
	2023			2018–2022		
	Mean HRI ED visit rate (95% CI)	Mean RR (95% CI)	p-value	Mean HRI ED visit rate (95% CI)	Mean RR (95% CI)	p-value
**Total**	**180 (155–208)**	**NA**	**NA**	**151 (128–177)**	**NA**	**NA**
**Age group, yrs**						
<18 (Ref)	95 (77–116)	NA	NA	85 (68–105)	NA	NA
18–25	211 (183–241)	2.52 (2.16–2.94)	<0.001	173 (148–201)	2.32 (2.00–2.69)	<0.001
26–54	222 (194–253)	2.54 (2.23–2.88)	<0.001	180 (155–208)	2.27 (2.01–2.57)	<0.001
55–64	207 (180–237)	2.29 (1.97–2.65)	<0.001	166 (142–193)	2.01 (1.74–2.33)	<0.001
65–74	173 (148–201)	1.95 (1.67–2.28)	<0.001	150 (127–176)	1.85 (1.58–2.16)	<0.001
≥75	120 (99–143)	1.47 (1.25–1.73)	<0.001	109 (90–131)	1.46 (1.24–1.72)	<0.001
**Sex**						
Female (Ref)	104 (85–126)	NA	NA	86 (69–106)	NA	NA
Male	271 (240–305)	2.73 (2.54–2.94)	<0.001	229 (200–261)	2.77 (2.57–2.98)	<0.001
**HHS region**						
1	69 (54–87)	1.36 (1.03–1.81)	0.029	92 (74–113)	1.39 (1.11–1.74)	0.004
2^¶^ (Ref)	51 (38–67)	NA	NA	66 (51–84)	NA	NA
3	121 (100–145)	2.43 (1.93–3.05)	<0.001	144 (121–170)	2.22 (1.84–2.68)	<0.001
4	226 (197–257)	4.58 (3.74–5.59)	<0.001	183 (157–212)	2.85 (2.42–3.37)	<0.001
5	102 (83–124)	2.03 (1.63–2.53)	<0.001	109 (90–131)	1.67 (1.40–2.01)	<0.001
6	483 (441–528)	9.89 (8.05–12.15)	<0.001	254 (224–287)	4.00 (3.33–4.80)	<0.001
7	327 (293–364)	6.60 (5.02–8.68)	<0.001	248 (218–281)	3.88 (2.99–5.04)	<0.001
8	127 (106–151)	2.47 (1.81–3.37)	<0.001	120 (99–143)	1.80 (1.36–2.38)	<0.001
9	298 (265–334)	5.92 (4.77–7.35)	<0.001	247 (217–280)	3.82 (3.16–4.60)	<0.001
10	128 (107–152)	2.53 (1.96–3.26)	<0.001	131 (110–155)	1.99 (1.59–2.48)	<0.001
**Peak heat season**						
Jul and Aug	303 (270–339)	3.07 (2.85–3.30)	<0.001	208 (181–238)	1.84 (1.72–1.97)	<0.001
Other warm-season months (May, Jun, and Sep) (Ref)	97 (79–118)	NA	NA	112 (92–135)	NA	NA

**Abbreviations:** ED = emergency department; HHS = U.S. Department of Health and Human Services; HRI = heat-related illness; NA = not applicable; Ref = referent group; RR = rate ratio.

* To reduce artifactual impact from changes in reporting patterns, analyses were restricted to facilities with a coefficient of variation for ED visits ≤40 and average weekly informative discharge diagnosis ≥70% complete with discharge diagnosis code formatting during January 2018–December 2023. After applying this data quality filter, a maximum of 823 ED (range = 3–823; median = 111) facilities that participate in the National Syndromic Surveillance Program returned one or more visits associated with HRI. https://www.cdc.gov/nssp/index.html

^†^ HRI ED visits per 100,000 ED visits.

^§^ https://www.hhs.gov/about/agencies/iea/regional-offices/index.html

^¶^ Region 2 (Ref) includes New Jersey, New York, Puerto Rico, and the U.S. Virgin Islands. Puerto Rico and the U.S. Virgin Islands currently do not report data to the National Syndromic Surveillance Program.

### Demographic Characteristics of Persons with HRI ED Visits

In 2023, among the demographic groups considered, higher rates of HRI ED visits were observed among males (271 per 100,000 ED visits) than among females (104) and among adults aged 18–64 years (range = 207–222) than adults aged ≥65 years (range = 120–173). In addition, the risk for HRI ED visits among adults aged 18–25 and 26–54 years was approximately 2.5 times the risk in the referent population (persons aged <18 years).

### Regional Differences in HRI ED Visits

HHS regional differences in warm-season HRI ED visit rates were observed in 2023. The lowest average warm-season HRI ED visit rate (51 per 100,000 ED visits) was reported by HHS Region 2 (New Jersey and New York), whereas the highest rate was reported by Region 6 (Arkansas, Louisiana, New Mexico, Oklahoma, and Texas) (483). Compared with Region 2 (the referent region), the HRI ED visit risks for regions 4, 6, 7, and 9 in 2023 were 1.5–2.5 times those during 2018–2022.

Daily HRI ED visit rates during the warm-season months in 2023 for several regions exceeded the 95th percentile of the daily HRI ED visit rate distribution for the warm-season months during 2018–2022 for multiple periods of ≥3 consecutive days in some regions (Supplementary Figure, https://stacks.cdc.gov/view/cdc/153146). For instance, in regions 6 and 9, HRI ED rates in July 2023 exceeded the 2018–2022 95th percentile for 16 and 18 consecutive days, respectively. In the warm-season months of 2023, every HHS region experienced ≥1 day above the 95th percentile (Table 2). In regions 4, 6, 7, and 9, the number of days with HRI ED visit rates exceeding the 95th percentile was higher than that in any previous year in the study period. In Region 6 alone, more than one third (37%; 56) of the days during the warm season of 2023 had daily HRI ED visit rates exceeding the 95th percentile. Regions 6 and 7 experienced days with the highest rate of HRI ED visits ever recorded in the ESSENCE system for their respective region since 2018.

**TABLE 2:** Number of days that the heat-related illness emergency department visit rate exceeded the 95th percentile,* by U.S. Department of Health and Human Services region, month, and year — United States, 2018–2023^†^

HHS region^§^/Month	No. of days, by year					
	2018	2019	2020	2021	2022	2023
**Region 1**						
May	0	0	0	0	0	0
Jun	1	0	1	7	1	0
Jul	5	4	4	0	5	3
Aug	5	0	0	3	2	0
Sep	0	0	0	0	0	0
**Region 2** ^¶^						
May	0	0	0	0	2	0
Jun	1	0	0	5	1	0
Jul	4	4	5	2	5	4
Aug	2	0	0	3	3	0
Sep	1	0	0	0	0	0
**Region 3**						
May	0	0	0	0	2	0
Jun	2	1	0	5	1	0
Jul	4	6	5	1	3	4
Aug	2	1	0	2	2	0
Sep	1	0	0	0	0	1
**Region 4**						
May	0	2	0	0	0	0
Jun	2	0	0	0	8	3
Jul	2	10	6	2	5	18
Aug	0	1	0	0	0	14
Sep	0	0	0	0	0	0
**Region 5**						
May	2	0	1	0	2	0
Jun	5	2	0	2	5	0
Jul	3	8	6	0	0	2
Aug	0	0	0	1	1	3
Sep	0	0	0	0	0	0
**Region 6**						
May	0	0	0	0	0	0
Jun	0	0	0	1	10	13
Jul	4	3	3	0	13	17
Aug	0	2	2	0	0	23
Sep	0	0	0	0	0	3
**Region 7**						
May	0	0	0	0	0	0
Jun	6	2	1	4	7	2
Jul	4	5	0	4	3	5
Aug	0	1	0	0	1	8
Sep	0	0	0	0	0	0
**Region 8**						
May	0	0	0	0	0	0
Jun	2	0	0	9	2	0
Jul	3	2	1	7	7	11
Aug	1	0	2	0	0	0
Sep	0	0	0	0	2	0
**Region 9**						
May	0	0	0	0	0	0
Jun	0	1	0	6	2	0
Jul	3	2	3	6	5	21
Aug	0	0	6	0	0	0
Sep	0	0	2	0	2	0
**Region 10**						
May	0	0	0	0	0	1
Jun	0	1	0	8	3	0
Jul	6	0	1	4	8	3
Aug	3	0	0	2	2	5
Sep	0	0	0	0	0	0

**Abbreviations:** ED = emergency department; HHS = U.S. Department of Health and Human Services.

* 95th percentile based on region-specific heat-related illness ED visit rate during warm-season months (May–September) during 2018–2022.

**^†^** To reduce artifactual impact from changes in reporting patterns, analyses were restricted to facilities with a coefficient of variation for ED visits ≤40 and average weekly informative discharge diagnosis ≥70% complete with discharge diagnosis code formatting during January 2018–December 2023. After applying this data quality filter, a maximum of 823 ED (range = 3–823; median = 111) facilities that participate in the National Syndromic Surveillance Program returned one or more visits associated with heat-related illness. https://www.cdc.gov/nssp/index.html

^§^ https://www.hhs.gov/about/agencies/iea/regional-offices/index.html

^¶^ Region 2 includes New Jersey, New York, Puerto Rico, and the U.S. Virgin Islands. Puerto Rico and the U.S. Virgin Islands currently do not report data to the National Syndromic Surveillance Program.

## Discussion

In recent years, health emergencies caused by heat exposure have become more frequent and widespread in the United States (*1*). The severity, frequency, and duration of heat waves in 2023 in some HHS regions resulted in record-high rates of HRI ED visits during the year, which prompted CDC to issue Epidemic Information Exchange (Epi-X) public health alerts.^§§§^

The finding of increased risk for HRI ED visit rates among certain demographic groups in 2023, particularly among males and adults aged 18–64 years, is similar to findings reported in other studies (*3*). Although the lowest HRI ED visit rates occurred among persons aged <18 years, previous studies of children and adolescents in different age groups suggest that children might also be subject to the effects of heat exposure at rates similar to those among adults in some areas of the United States (*4*). Persons who work outdoors might regularly endure extreme heat; this group warrants particular attention because of the high prevalence of HRI ED visits observed in working-aged adults. Frontline essential workers tending to emergencies, such as firefighters, might be at particularly high risk for exposure to heat stress (*5*). Regional differences in rates of HRI ED visits might reflect differential acclimatization, behavioral responses, and adaptation strategies (*1*,*6*). Understanding the causes of these differences can help guide the development and implementation of public health interventions, such as heat action plans and issuance of heat alerts calibrated based on local epidemiologic data (e.g., HeatRisk).^¶¶¶^

Effective implementation of heat mitigation strategies is associated with social determinants of health. For example, even in areas with high rates of air conditioning, such as the South and southeastern United States, persons exposed to extreme heat might have limited or no access to cooling spaces (*1*). Factors that affect air conditioning use and access to cooling spaces include energy costs**** and the occurrence of outages due to power grid failure (*1*,*7*,*8*). HHS programs that provide financial assistance for residential energy^††††^ and monitor the safety of persons reliant on electricity-dependent durable medical equipment in case of power outages during extreme heat^§§§§^ can protect populations affected by heat stress. The intersection of communities with a high proportion of groups at risk, especially those with limited access to health care, with areas that experience persistent high ambient temperatures (e.g., heat islands or lack of green spaces) could be more susceptible to the effects of heat exposure (*1*). Public health initiatives can be designed to help communities prepare for extreme heat conditions and complement the efforts of weather and emergency management agencies, reducing illnesses and deaths. Tools used for syndromic surveillance, including ESSENCE, local systems, and visualization dashboards, help guide and strengthen public health preparedness and response. An example is CDC's Heat and Health Tracker (https://ephtracking.cdc.gov/Applications/heatTracker/), which provides local heat and health information for communities.

### Limitations

The findings in this report are subject to at least five limitations. First, NSSP data are not nationally representative, and participation can vary by HHS region. Second, although the prevalence of HRI among U.S. military veterans has been increasing (*9*), this analysis does not include facilities operated by U.S. Department of Veterans Affairs. In addition, the HRI ED visit rate reported by ESSENCE might not be representative of the rate in the general population because ESSENCE is not a population-based system but rather reflects the number of HRI ED visits among all-cause ED visits. Third, HRI information reported at the HHS regional level can obscure subregional variation. Fourth, estimation of HRI ED visit rates might have been affected during the COVID-19 pandemic because overall ED utilization patterns changed for specific subpopulations (*10*). Finally, HRI data from the ESSENCE system are based on ED visits only and do not identify cases of HRI among persons who sought treatment elsewhere, likely resulting in an underestimation of HRI prevalence.

### Implications for Public Health Practice

The record-breaking temperatures of the 2023 warm-weather season had a substantial public health impact, and this trend might increase in the coming years because of climate change (*1*). Public health agencies rely on tools and surveillance systems to assess the adverse health effects of heat exposure. Timely mechanisms for tracking and reporting health effects, along with the ability to detect anomalous trends, especially during extreme heat emergencies, can facilitate the implementation of public health strategies to protect affected populations.

## Footnotes

*
https://www.nasa.gov/news-release/nasa-announces-summer-2023-hottest-on-record/

†
https://www.whitehouse.gov/briefing-room/statements-releases/2023/07/27/fact-sheet-president-biden-to-announce-new-actions-to-protect-workers-and-communities-from-extreme-heat

§
https://www.cdc.gov/disasters/extremeheat/index.html

¶ HRI ED visits were identified using administrative discharge diagnosis codes and free text search of the patient’s reason for visit (i.e., their chief complaint). https://knowledgerepository.syndromicsurveillance.org/heat-related-illness-v2

**
https://publichealth.jmir.org/2021/6/e26303/authors

†† The dataset does not include data from American Samoa, Federated States of Micronesia, Marshall Islands, Northern Mariana Islands, Palau, Puerto Rico, or the U.S. Virgin Islands. https://www.hhs.gov/about/agencies/iea/regional-offices/index.html

§§ To reduce artifactual impact from changes in reporting patterns, analyses were restricted to facilities with a coefficient of variation for ED visits ≤40 and average weekly informative discharge diagnosis ≥70% complete with discharge diagnosis code formatting during January 2018–December 2023. After applying this data quality filter, a maximum of 823 ED (range = 3–823; median = 111) facilities that participate in NSSP returned one or more visits associated with HRI. https://www.cdc.gov/nssp/index.html

¶¶ 45 C.F.R. part 46.102(l)(2), 21 C.F.R. part 56; 42 U.S.C. Sect. 241(d); 5 U.S.C. Sect. 552a; 44 U.S.C. Sect. 3501 et seq.

*** All facilities reporting HRI data to NSSP were considered to obtain the total HRI ED visits reported during January 1–December 31, 2023, across all HHS regions. During this period, HRI data were reported by a maximum of 1,238 EDs (range = 12–1,238; median = 152) that participated in NSSP and returned one or more visits associated with HRI.

†††
https://www.ncei.noaa.gov/access/monitoring/monthly-report/global/202313

§§§ On June 30, 2023, and August 23, 2023, CDC issued public health alerts using the Epi-X system upon noticing high levels of heat-related ED visits.

¶¶¶ HeatRisk is a health-based heat forecast developed by the National Oceanic and Atmospheric Administration’s National Weather Service and CDC. It integrates health and temperature data to deliver a 7-day outlook for hot weather. HeatRisk uses a 5-level scale to indicate how risky the heat level is in a specific area. www.cdc.gov/HeatRisk

****
https://neada.org/wp-content/uploads/2023/07/summercoolingestPR.pdf

††††
https://www.acf.hhs.gov/ocs/programs/liheap

§§§§
https://empowerprogram.hhs.gov/
